# Local Colonic Administration of a Serine Protease Inhibitor Improves Post-Inflammatory Visceral Hypersensitivity in Rats

**DOI:** 10.3390/pharmaceutics13060811

**Published:** 2021-05-29

**Authors:** Nikita Hanning, Michelle De bruyn, Hannah Ceuleers, Tim Boogaerts, Maya Berg, Annemieke Smet, Heiko U. De Schepper, Jurgen Joossens, Alexander L. N. van Nuijs, Joris G. De Man, Koen Augustyns, Ingrid De Meester, Benedicte Y. De Winter

**Affiliations:** 1 Laboratory of Experimental Medicine and Pediatrics (LEMP), University of Antwerp, 2610 Wilrijk, Belgium; nikita.hanning@uantwerpen.be (N.H.); hannah.ceuleers@uantwerpen.be (H.C.); annemieke.smet@uantwerpen.be (A.S.); heiko.deschepper@uantwerpen.be (H.U.D.S.); joris.deman@uantwerpen.be (J.G.D.M.); 2 Infla-Med, Centre of Excellence, University of Antwerp, 2610 Wilrijk, Belgium; michelle.debruyn2@uantwerpen.be (M.D.b.); maya.berg@uantwerpen.be (M.B.); koen.augustyns@uantwerpen.be (K.A.); ingrid.demeester@uantwerpen.be (I.D.M.); 3 Laboratory of Medical Biochemistry, University of Antwerp, 2610 Wilrijk, Belgium; 4 Toxicological Centre, University of Antwerp, 2610 Wilrijk, Belgium; tim.boogaerts@uantwerpen.be (T.B.); alexander.vannuijs@uantwerpen.be (A.L.N.v.N.); 5 Department of Gastroenterology and Hepatology, Antwerp University Hospital (UZA), 2650 Edegem, Belgium; 6 Laboratory of Medicinal Chemistry, University of Antwerp, 2610 Wilrijk, Belgium; jurgen.joossens@uantwerpen.be

**Keywords:** elastase, irritable bowel syndrome, proteolytic activity, serine proteases, trypsin, visceral hypersensitivity

## Abstract

Dysregulation of the protease–antiprotease balance in the gastrointestinal tract has been suggested as a mechanism underlying visceral hypersensitivity in conditions such as inflammatory bowel disease (IBD) and irritable bowel syndrome (IBS). We aimed to study the potential therapeutic role of an intracolonically administered serine protease inhibitor for the treatment of abdominal pain in a post-inflammatory rat model for IBS. An enema containing 2,4,6-trinitrobenzene sulfonic acid (TNBS) was used to induce colitis in male Sprague–Dawley rats, whereas controls received a saline solution. Colonoscopies were performed to confirm colitis and follow-up mucosal healing. In the post-inflammatory phase, the serine protease inhibitor UAMC-00050 (0.1–5 mg/kg) or its vehicle alone (5% DMSO in H_2_O) was administered in the colon. Thirty minutes later, visceral mechanosensitivity to colorectal distensions was quantified by visceromotor responses (VMRs) and local effects on colonic compliance and inflammatory parameters were assessed. Specific proteolytic activities in fecal and colonic samples were measured using fluorogenic substrates. Pharmacokinetic parameters were evaluated using bioanalytical measurements with liquid chromatography–tandem mass spectrometry. Post-inflammatory rats had increased trypsin-like activity in colonic tissue and elevated elastase-like activity in fecal samples compared to controls. Treatment with UAMC-00050 decreased trypsin-like activity in colonic tissue of post-colitis animals. Pharmacokinetic experiments revealed that UAMC-00050 acted locally, being taken up in the bloodstream only minimally after administration. Local administration of UAMC-00050 normalized visceral hypersensitivity. These results support the role of serine proteases in the pathophysiology of visceral pain and the potential of locally administered serine protease inhibitors as clinically relevant therapeutics for the treatment of IBS patients with abdominal pain.

## 1. Introduction

Proteases are abundantly present in the gastrointestinal (GI) tract and play a vital role in the digestive process, as well as in preserving intestinal homeostasis [1]. Alterations in the expression or activity levels of several proteases and endogenous protease inhibitors are associated with GI diseases such as inflammatory bowel disease (IBD) [2,3,4,5] and irritable bowel syndrome (IBS) [5,6,7,8,9,10,11,12,13]. Dysregulation of the protease–antiprotease balance, leading to increased proteolytic activity, causes intestinal barrier dysfunction as well as visceral hypersensitivity in these conditions [14,15,16].

Activation of protease-activated receptors (PARs), degradation of tight junction proteins, interaction with adherence junctions, or potentiation of cytokines and chemokines underlies increased intestinal permeability and visceral hypersensitivity [1,12,14,17,18]. One interesting protease is elastase, demonstrating an increased activity in colonic tissue from IBD patients and fecal supernatant from a subset of post-infection IBS patients [13,19]. This activity can originate from neutrophil elastase or elastase A2, which was recently revealed to be produced by colonic epithelial cells [18,20]. Elastase interacts with E-cadherin, activates PAR2, and increases the expression of pro-inflammatory cytokines to cause the events mentioned above [1,19]. An intriguing set of enzymes contains the trypsin-like enzymes. Increased trypsin-like activity was found in colonic tissue from a post-inflammatory rat model for IBS and colonic tissue and from fecal supernatant from IBS patients [8,13,16,21]. Furthermore, trypsin-3 and tryptase both reduce the epithelial barrier function and cause visceral hypersensitivity by means of PAR2 activation [12,14].

The inhibition of proteases has been proposed as a therapeutic approach for the treatment of functional GI diseases [1,22], and several animal studies have shown promising effects of serine protease inhibitors on visceral pain [1,15] and intestinal permeability [1,14]. To our knowledge, no clinical trials have been performed to study the effect of serine protease inhibitors in these functional GI disorders. Considering the large spectrum of functions of proteases throughout the body, systemic delivery of protease inhibitors could theoretically be associated with side effects such as interference with blood coagulation or cell proliferation. The occurrence of these side effects could be limited when the drug would target specific serine proteases or could be delivered locally.

In a pivotal study, Motta and colleagues demonstrated that elafin could be delivered in the colon using lactic acid bacteria engineered to express this endogenous protease inhibitor, resulting in decreased inflammation and barrier dysfunction in mouse models of acute and chronic colitis [19]. However, whether local delivery of protease inhibitors can affect visceral pain remains unknown.

Because of the role of serine proteases in inflammation and GI diseases, we aimed to characterize them in colonic and fecal samples from a rat post-colitis model using fluorogenic substrates. It is of great interest to determine the protease activity, since the mRNA or protein expression levels of the enzymes might not reflect their activity [23]. Furthermore, we aimed to investigate the in vivo effects of an intracolonically administered serine protease inhibitor on colonic and fecal proteolytic activity and hypothesized that decreased proteolytic activity would be accompanied by a reduction in visceral hypersensitivity. Our study was conducted using the serine protease inhibitor UAMC-00050, since this compound is known to have beneficial effects on post-inflammatory visceral hypersensitivity in rats after systemic delivery [16]. Moreover, this drug shows an auspicious inhibitory profile with a good inhibition of tryptase, as well as a limited inhibition of proteases related to the coagulation cascade [16].

## 2. Materials and Methods

### 2.1. Animals

Adult male Sprague–Dawley rats (150–175 g) were purchased from Charles River Laboratories (Italy) and used for all animal experiments. Animals were housed at constant room temperature (22 ± 2 °C) and humidity (60%) on a 12-h light/dark cycle (7:00 a.m.–7:00 p.m.) with two rats per cage and unlimited access to water and food. Rats were allowed to acclimatize to the animal facility for 5–10 days prior to experimental procedures. All experiments were approved by the Ethical Committee for Animal Experiments of the University of Antwerp (EC nr. 2018-79).

Sample size calculations were performed to determine the number of animals necessary to have sufficient statistical power (α = 0.05, 1 − β = 0.80). Differences in colonic proteolytic activity and visceral sensitivity between UAMC-00050-treated animals and vehicle-treated animals were considered to be primary endpoints. To detect differences in colonic proteolytic activity, 12 animals per group were necessary to have sufficient power. To detect differences in visceral sensitivity, 8 animals per group were necessary to have sufficient power.

Based on the sample size calculations, we included 8 animals per group for measuring visceral sensitivity, colonic compliance, assessing the inflammatory parameters, and collecting colonic tissue and fecal samples. To obtain sufficient power to detect differences in proteolytic activity, an additional 4 animals per group were included specifically for tissue harvesting. Pharmacokinetic experiments were performed on a separate group of rats (*n* = 12 in total).

### 2.2. Experimental Design

**Figure 1:**
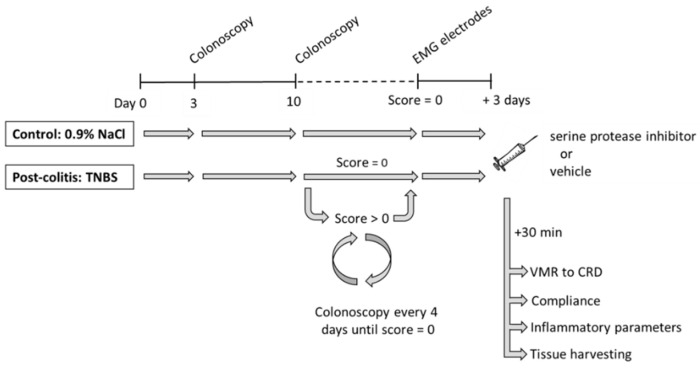
Schematic overview of the experimental design. After induction of distal colitis with an enema containing 2,4,6-trinitrobenzenesulfonic acid (TNBS), the course of colitis was monitored endoscopically on day 3 to confirm the presence of and assess the severity of colitis, and then every 4 days, starting from day 10, until full mucosal healing was observed. Then, electromyographic (EMG) electrodes were implanted. Three days later, animals received an enema containing the serine protease inhibitor UAMC-00050 or its vehicle (5% DMSO). Further experiments were conducted sequentially, 30 min later. CRD, colorectal distensions; EMG, electromyographic; TNBS, 2-4-6-trinitrobenzenesulfonic acid; VMR, visceromotor response.

A well-characterized 2,4,6-trinitrobenzene sulfonic acid (TNBS)-induced post-colitis rat model for visceral hypersensitivity was used in this study [16,24,25]. The experimental course is shown in Figure 1. Details on the procedures are given in subsequent paragraphs. After acclimation, an enema containing TNBS was used to induce a mild colitis, whereas control rats received an enema containing saline. The presence and severity of acute colitis were scored colonoscopically 3 days later. Subsequently, a colonoscopy was performed starting from day 10 and repeated every 4 days until complete mucosal healing was observed. Then, electromyographic (EMG) electrodes were implanted in the abdominal musculature of the rat and exteriorized at the scapular region. Three days later, the serine protease inhibitor UAMC-00050 or its vehicle was given intracolonically. Thirty minutes after the single administration of the drug, visceral sensitivity to colorectal distensions and colonic compliance were evaluated. Finally, animals were sacrificed (exsanguination under pentobarbital anesthesia, 45 mg/kg i.p.) and inflammatory parameters (colonoscopy, macroscopy, microscopy, and myeloperoxidase (MPO) activity) were scored to confirm the post-inflammatory state of the animal and to evaluate effects of the compound on mucosal inflammation. Colonic and fecal samples were taken for the assessment of proteolytic activities using selective fluorogenic substrates. Pharmacokinetic experiments were performed in a separate group of rats.

### 2.3. Materials and Reagents

The following reagents were used: Eosin and 100% ethanol (Acros Organics, Geel, Belgium); fluorogenic substrates Boc-Gln-Ala-Arg-AMC, n-Tosyl-Gly-Pro-AMC, Suc-Ala-Ala-Pro-Phe-AMC, Suc-Ala-Ala-Pro-Val-AMC, Suc-Ala-Ala-Ala-AMC, and H-Pro-Phe-Arg-AMC (Bachem, Bubendorf, Switzerland); xylazine 2% (Rompun^®^, Bayer, Leverkussen, Germany); acetonitrile (LC-MS grade) (BioSolve, Valkenswaard, The Netherlands); sodium chloride solution (0.9%) (Braun, Diegem, Belgium); pentobarbital 60 mg/mL (Nembutal^®^; Ceva, Brussels, Belgium); methanol (LC-MS grade) (Fisher Scientific, Loughborough, UK); formaldehyde, formic acid (98–100%) and hematoxylin (Merck, Darmstadt, Germany); cathepsin G inhibitor I, heparin, octylglucoside and TNBS (Sigma-Aldrich, Overijse, Belgium); ketamine 100 mg/mL (Ketalar^®^; Pfizer, Brussels, Belgium); UAMC-00050 (lab of medicinal chemistry, University of Antwerp, Antwerp, Belgium) [26,27]; Type 1 (ultrapure) water (18.2 MΩ.cm) (Purelab, ELGA Veolia, High Wycombe, UK).

The Krebs–Ringer solution was prepared in house and had the following composition: 118.3 mM NaCl, 4.7 mM KCl, 2.5 mM CaCl_2_, 2 mM NaHCO_3_, 1.2 mM KH_2_PO_4_, 1.2 mM MgSO_4_, 0.026 mM CaEDTA, and 11.1 mM glucose (all compounds from Merck, Darmstadt, Germany).

For the VMR experiments, a fresh solution of UAMC-00050 was prepared from the powder by dissolving in 5% DMSO in H_2_O. The concentration of the compound necessary to ensure delivery of the correct dose (0.1–1.0–2.5–5.0 mg/kg) was adapted to a fixed volume of 200 µL.

For pharmacokinetic experiments, a stock solution of UAMC-00050 at 1 mg/mL was prepared from the powder by dissolving in methanol; further working solutions were diluted from the stock solution in methanol. The internal standard (IS) nordiazepam-D_5_ was obtained as a solution (1 mg/mL in methanol) from Cerilliant Corporation (Round Rock, TX, USA). The IS working solution was prepared by diluting the stock solution in methanol. The stock and the working solutions for both UAMC-00050 and nordiazepam-D_5_ were stored at −20 °C.

### 2.4. Induction of Colitis

After an overnight fast with free access to water, rats were anesthetized with a mixture of ketamine (35 mg/kg, i.p.) and xylazine (5 mg/kg, i.p.) and placed in a Trendelenburg position. A flexible catheter (18 G, length 4.5 cm) was used to administer a 250 µL TNBS-enema (4 mg in 50% ethanol) or saline in the distal colon of randomized colitis and control rats, respectively, according to previously published protocols [16,24]. Afterwards, animals were allowed to recover in their own cages with free access to water and food.

### 2.5. Intracolonic Drug Administration

The serine protease inhibitor UAMC-00050 (0.1–1.0–2.5–5.0 mg/kg, dissolved in a volume of 200 µL) or its vehicle (5% DMSO in H_2_O) was delivered colonically using a flexible catheter (18 G, length 4.5 cm) under short-lasting isoflurane anesthesia (5.0% induction, 1.5% maintenance). To prevent expulsion of the enema, animals were held in a Trendelenburg position for 90 s. Doses for intracolonic administration were based on the doses known to be effective via an i.p. administration [16] and adapted afterwards based on preliminary results.

### 2.6. Colonic Mechanosensitivity

Contractions of the abdominal musculature in response to isobaric colorectal distensions, a nociceptive reflex known as the visceromotor response (VMR), were used to quantify colonic mechanosensitivity in vivo [24,28]. To this end, 2 Teflon-coated EMG electrodes (AISI 316 stainless steel, ø 0.125 mm, Advent Research Material Ltd., Oxford, UK) were sutured into the abdominal external oblique muscle, in close proximity to the right inguinal ligament and 5 mm apart. Then, the electrodes were tunneled, subcutaneously towards the base of the neck, exteriorized, and fixated with a plastic cap for future access. Rats were single-housed during their 3-day recovery after the surgical procedure to protect the electrodes and plastic cap from damage.

On the day of the VMR experiment, a lubricated balloon (length 5.0 cm) was introduced into the colorectum of the conscious rat and connected to a barostat, allowing phasic isobaric colonic distensions (Distender Series IIR Barostat, G&J Electronics, Toronto, ON, Canada). EMG electrodes were attached to a data acquisition system and a standardized, pressure-controlled phasic distension protocol was used to expand the balloon (20 s distensions of 10–20–30–40–60 mmHg, 4 min interval). The EMG-signal was registered (NL100AK AC headstage, Digitimer Ltd., Letchworth, UK), amplified (gain 2k, NL104, Digitimer Ltd., Letchworth, UK), and digitized (CED 1401, Cambridge Electronic Design, Cambridge, UK). EMG recordings were analyzed using Spike2 Version 5.07 (Cambridge Electronic Design, Cambridge, UK). The magnitude of the VMR signal was quantified at each distension pressure using the integral of the EMG signal during the distension (area under the curve, AUC; expressed as µV/20 s) and normalized by subtracting the AUC of the EMG signal before the distension.

### 2.7. Colonic Compliance

Colonic compliance is a measure for the resistance of the colon against deformation and is usually expressed as the difference quotient of a volume-pressure curve of the colon [29]. Since compounds can alter the VMR by acting either directly on the pain signaling pathways or on the viscoelastic properties of the colon, colonic compliance was evaluated in the rats. First, the rat was placed in a supine position. A lubricated balloon (length 5.0 cm) was inserted into the colorectum under pentobarbital anesthesia (45 mg/kg, i.p.). Then, the balloon was filled with increasing fixed volumes of water (0.0–0.5–1.0–1.5–2.0 mL, 80 s interval) while the corresponding intracolonic pressure was measured using a pressure transducer (Gould P23-ID Statham), amplified (gain 1 V/100 mHg, NL108 A, Digitimer Ltd., Letchworth, UK) and digitized (CED 1401, Cambridge Electronic Design, Cambridge, UK) before it was analyzed using Spike 2 (version 5.07). Pressure-volume curves were used to represent colonic compliance.

### 2.8. Inflammatory Parameters

#### 2.8.1. Colonoscopy

An in vivo colonoscopy was performed to assess the presence and extent of TNBS-induced colitis, as previously described [24,30]. Briefly, rats were anesthetized (ketamine/xylazine, 35/5 mg/kg, i.p.) and placed in a supine position. The lubricated tip of a pediatric upper gastrointestinal endoscope (Olympus GIF-N30) was carefully introduced into the colorectum and advanced until the hepatic flexure was reached. Then, the endoscope was slowly withdrawn while assessing the mucosal damage using a validated and standardized scoring system (Supplementary Table S1). In brief, a total inflammatory score (0–19) was calculated based on the extent of ulcerations, as well as on the presence of edema, bleeding, and colonic stenosis [30].

#### 2.8.2. Macroscopic Score

After VMR and compliance measurements, the rat was sacrificed by exsanguination under pentobarbital anesthesia (45 mg/kg, i.p.). The colon was excised, rinsed with Krebs solution, opened along the mesenteric border, and evaluated visually for the presence of mucosal damage using a previously validated scoring system (total score 0–10, Supplementary Table S2) [30].

#### 2.8.3. Microscopic Score

For microscopic evaluation, a representative section of the distal colon (1 cm^2^) was fixed in 4% formaldehyde for 24 h and embedded in paraffin for hematoxylin and eosin staining. Transverse sections (5 µm) were scored for the presence of inflammatory infiltrate and alterations in mucosal architecture, as previously described (total score 0–10, Supplementary Table S3) [30].

#### 2.8.4. Myeloperoxidase Activity

Finally, colonic MPO activity was determined in a full-thickness colonic segment (±30 mg) as published previously [30]. MPO is a pro-inflammatory enzyme stored in the azurophilic granules of neutrophilic granulocytes and monocytes. Hence, the tissue MPO activity is a measure of the myeloid cell infiltration of the colonic wall [31]. One unit (U) of MPO activity is defined as the amount of enzyme required to convert 1 µmol H_2_O_2_ to H_2_O within 1 min at 25 °C and is expressed as U/g tissue [31].

### 2.9. Proteolytic Activities on Fecal and Colonic Samples

Trypsin-like, chymotrypsin-like, cathepsin G, elastase-like, and kallikrein activities were determined in colon tissue and fecal samples to profile serine protease activity. Immediately after the rat was sacrificed, distal colon samples were taken, rinsed with Krebs solution, snap-frozen, and immediately stored at −80 °C until further processing. Fecal samples were taken before treatment with UAMC-00050 (5 mg/kg) and stored at −80 °C as well. Colonic tissue and feces were crushed in a pre-cooled mortar (−80 °C) on dry ice to prevent enzymatic activity loss due to temperature increase. Liquid nitrogen was poured over the samples after each crushing step. The obtained powder from the colon samples was lysed for 15 min, the fecal samples were lysed for 10 min. Afterwards, colonic and fecal samples were centrifuged in a pre-cooled centrifuge (4 °C 12,000× *g*) for 5 and 10 min, respectively. The composition of the lysis buffer depended on the sample type and activity assay. Colon tissue was lysed in a buffer containing 1% octylglucoside in 50 mM Tris-HCl pH 7.4, and the same buffer without octylglucoside was used for the fecal samples. Furthermore, 0.05% heparin and 0.1% heparin were added to the lysis buffers to assess chymotrypsin- and trypsin-like activity, respectively.

Boc-Gln-Ala-Arg-AMC (75 µM) and n-Tosyl-Gly-Pro-Arg-AMC (100 µM) in 50 mM Tris-HCl pH 8.0 containing 120 mM NaCl were used to measure the trypsin-like activity. Samples were placed in a cold microtiter plate and preheated substrate (37 °C) was added to start the reaction. The fluorescence was measured during 20 min at 37 °C on a Tecan Infinite F200 Pro microtiter plate reader. The other activities were assessed in a similar assay, using the substrates mentioned below. Chymotrypsin-like and kallikrein activity were measured with Suc-Ala-Ala-Pro-Phe-AMC (0.45 mM) and H-Pro-Phe-Arg-AMC (0.45 mM), respectively. Elastase-like activity was measured using two assays, one using Suc-Ala-Ala-Pro-Val-AMC as a substrate and the other using Suc-Ala-Ala-Ala-AMC (both 0.45 mM). Cathepsin G activity was measured with Suc-Ala-Ala-Pro-Phe-AMC by making the difference in activity without and with cathepsin G inhibitor I.

### 2.10. Pharmacokinetic Experiments

To evaluate the pharmacokinetic properties of UAMC-00050, it was administered in male Sprague–Dawley rats (200–225 g) either colonically (5 mg/kg, in a volume of 200 µL 5% DMSO solution) or intravenously (1 mg/kg in a volume of 200 µL 5% DMSO solution). Plasma samples were collected after 5, 15, 30, 45, 60, 90, 120, and 180 min via the tail vein and stored at −80 °C until analysis.

Sample preparation and analysis of plasma samples consisted of protein precipitation followed by direct injection in a liquid chromatography–tandem mass spectrometry (LC-MS/MS) instrument. Briefly, 100 µL of plasma was spiked with 20 µL of nordazepam-D_5_ used as internal standard for quantification (yielding in a final concentration of 485 ng/mL) and afterwards 180 µL of acetonitrile was added to precipitate proteins. Subsequently, the samples were vortexed for 2.5 min at 2000× *g* and centrifuged for 5 min at 10,000× *g*. In a next step, 100 µL of the supernatant was transferred to a 0.20 µm centrifugal filter to remove remaining particles. After centrifugation for 5 min at 10,000× *g*, the remaining filtrate was transferred to an autosampler vial ready to be analyzed with LC-MS/MS. A seven-level point calibration curve with final concentrations ranging between 5 and 1000 ng/mL and a curve weighting factor of 1/x^2^ was prepared in blank mouse plasma using different working solution standards of UAMC-00050 and a fixed amount of internal standard. The lower limit of quantification of the method was 5 ng/mL.

Chromatographic separation was achieved with a Kinetex Biphenyl column (100 × 2.1 mm, 2.6 µm) on an Agilent 1260 LC system using a mobile phase composed of (A) 0.1% *v*/*v* formic acid in ultrapure water and (B) 10/90 ultrapure water/acetonitrile +0.1% *v*/*v* formic acid, at a flow rate of 0.3 mL/min. A gradient was applied as follows: 0–0.5 min: 5% B; 0.5–10 min 5–100% B; 10–12 min 100% B; and 12–12.1 min 5% B. The total run time was 18 min and the injection volume was set to 5 µL. Detection and quantification of UAMC-00050 were performed with an Agilent 6410 triple quadrupole MS system with an electrospray interface (ESI) operating in positive ionization mode. The following source parameters were applied: gas temperature 350 °C, gas flow 10 L/min, nebulizer 35 psi, and capillary voltage 4000 V. Compound-dependent MS parameters are summarized in Table 1. The most abundant transition of UAMC-00050 was used as quantifier and the other transitions were used for confirmation (qualifiers). For compound confirmation, the following criteria were used: the quantifier/qualifier ratio should fall within ±15% of the ratio observed in calibrators and the relative retention time should fall within 2.5% of the values observed in the calibrators.

### 2.11. Data Presentation and Statistical Analysis

All data are presented as mean ± SEM or as median (IQR). Pharmacokinetic analysis and modeling of the plasma concentration–time data was performed using PKSolver [32]. Other statistical analysis and data visualization processes were performed using R software (version 4.0.3) and GraphPad Prism (version 7.04). The nominal significance level was 0.05 for all tests. The development of body weight, VMR results, and colonic compliance were assessed with generalized estimating equations (GEE) using an unstructured working correlation matrix, followed by Fisher’s least significant difference (LSD) post hoc test on prespecified pairwise comparisons of interest. Inflammatory parameters were analyzed using two-way analysis of variance (ANOVA), followed by Student–Newman–Keuls (SNK) post hoc testing when applicable. To evaluate the differences in proteolytic activities between control, post-colitis, treated, and untreated rats, a two-way ANOVA was conducted. Main effects were tested on split files with a Mann–Whitney U test with Bonferroni correction. To assess correlations between specific fecal or colonic proteolytic activities and inflammatory parameters, the pairwise comparisons of interest were determined a priori. Spearman’s rank order correlation coefficient was used and the Holm–Bonferroni method was applied to correct for multiplicity.

## 3. Results

### 3.1. TNBS-Induced Colitis Resolves Spontaneously after 10–14 Days

**Figure 2:**
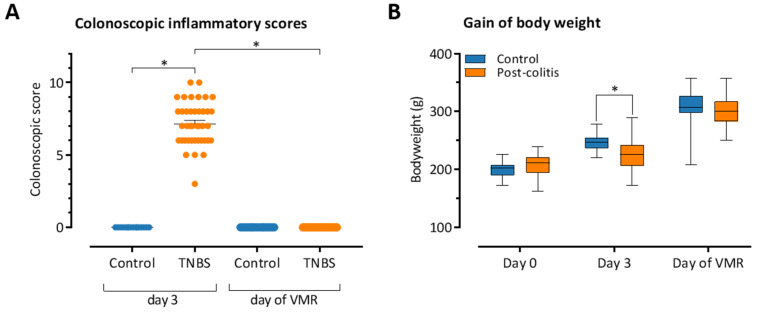
Evaluation of inflammatory parameters in the post-colitis rat model for visceral hypersensitivity. (**A**) Inflammatory colonoscopic scores on day 3 and on the day of the VMR experiments. Data are presented including mean ± SEM. Two-Way ANOVA with ‘treatment’ and ‘group’ factors followed by a Student–Newman–Keuls post hoc test. Significant effect of ‘group’ factor, but not ‘treatment’ factor. No significant interaction between ‘treatment’ and ‘group’; *n* = 8 per group, treatment groups were combined for visualization. (**B**) Increase in body weight during the experiment. Generalized estimating equations applied using an unstructured working correlation matrix, followed by a least-significant difference (LSD) *post hoc* test; *n* = 8 per group, treatment groups were combined for visualization. Note: * *p* < 0.05; significantly different.

Animals that were given an enema containing TNBS developed distal colitis, as demonstrated by the significantly higher colonoscopic inflammatory scores at day 3 compared to the control animals (7.15 ± 0.24 (*n* = 40) versus 0.00 ± 0.00 (*n* = 16), *p* < 0.001, pooling of different treatment groups, Figure 2A). Additionally, TNBS-treated rats gained less weight in the 3 days following the enema compared to their healthy counterparts (20.1 ± 2.7 g (*n* = 40) versus 45.9 ± 1.5 g (*n* = 16), *p* = 0.012, pooling of different treatment groups, Figure 2B).

After 10–14 days, colonic inflammatory scores normalized and were similar to those of controls, indicating that the colitis had resolved spontaneously and that the TNBS-treated rats reached the post-inflammatory state (post-colitis rats). Body weight in post-colitis rats was similar to control animals 3 days after full mucosal healing was observed, coinciding with the day of the VMR experiments (302.4 ± 4.3 g (*n* = 40) versus 306.2 ± 8.1 g (*n* = 16), *p* = NS, pooling of different treatment groups).

Drug allocation during the experiments was randomized for all rats. We did not observe significant differences in the clinical course of the colitis (e.g., colonoscopic inflammatory scores at day 3, duration of the colitis, and gradual increases in body weight) between the post-colitis animals that were allocated to different treatment groups (Table 2).

### 3.2. Colonic Trypsin-Like Activity Is Increased in Post-Colitis Rats

**Figure 3:**
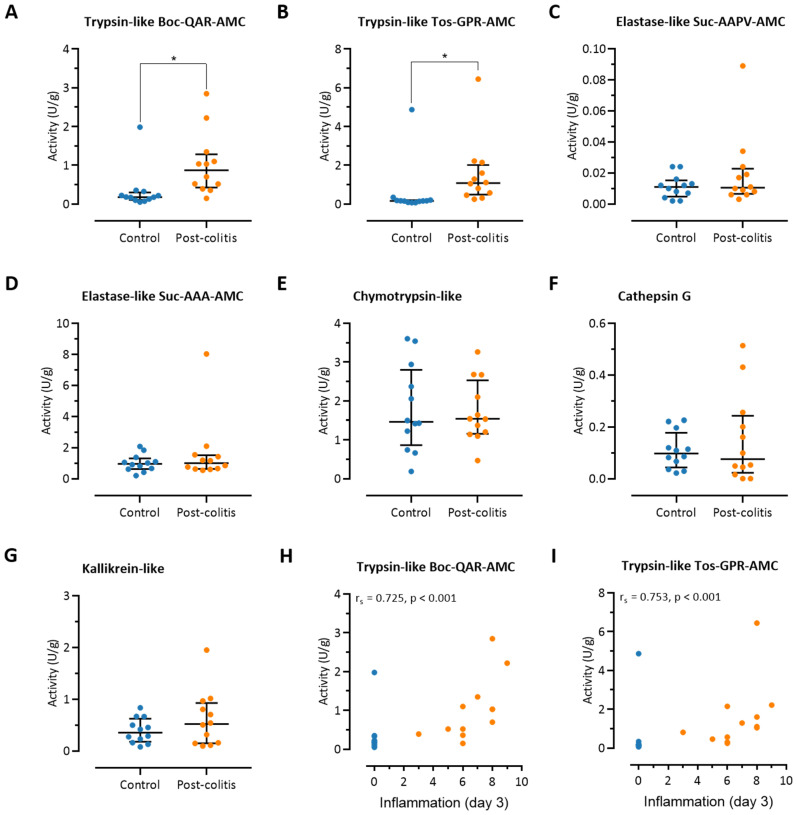
Proteolytic activities in colonic tissue of post-colitis and control rats. (**A**) Trypsin-like activity assessed using the substrate Boc-Gln-Ala-Arg-AMC. (**B**) Trypsin-like activity assessed using the substrate Tos-Gly-Pro-Arg-AMC. (**C**) Elastase-like activity assessed using the substrate Suc-Ala-Ala-Pro-Val-AMC. (**D**) Elastase-like activity assessed using the substrate Suc-Ala-Ala-Ala-AMC. (**E**) Chymotrypsin-like activity. (**F**) Cathepsin G activity. (**G**) Kallikrein activity. Proteolytic activities were analyzed using two-way ANOVA with ‘treatment’ and ‘group’ factors, followed by Mann–Whitney U tests with Bonferroni correction for multiplicity; *n* = 12 per group. Analysis was performed on the full dataset (involving the 4 combinations of control/post-colitis and vehicle/UAMC-00050), however visualization is split across Figure 3 and Figure 6, Figure S1 to allow for easier interpretation of the data. Data are presented including the median and IQR. (**H**) Correlation between the severity of inflammation in the acute phase of colitis (day 3) and trypsin-like activity (Boc-Gln-Ala-Arg-AMC substrate). (**I**) Correlation between the severity of inflammation in the acute phase of colitis (day 3) and trypsin-like activity (Tos-Gly-Pro-Arg-AMC substrate). Correlations were assessed using Spearman’s rank order correlation coefficient, employing the Holm–Bonferroni method to correct for multiplicity. Note: * *p* < 0.05; significantly different from control.

Trypsin-like activity was significantly increased in full-thickness samples taken from the distal colon of post-colitis rats in two assays assessing the cleavage of two trypsin substrates, Boc-Gln-Ala-Arg-AMC and Tos-Gly-Pro-Arg-AMC, compared to healthy controls (0.87 (0.49–1.16) U/g versus 0.18 (0.25–0.12) U/g, *p* < 0.01 and 1.08 (0.54–1.73) U/g versus 0.16 (0.13–0.19) U/g, *p* < 0.01, respectively, *n* = 12 per group, Figure 3A,B). Chymotrypsin-like, cathepsin G, elastase-like, and kallikrein activities were comparable in post-colitis and control animals (Figure 3C–G). Interestingly, the trypsin-like activity in the colonic tissue strongly correlated with the severity of inflammation in the acute phase of colitis at day 3 (r_s_ = 0.725, *p* < 0.001 for the Boc-Gln-Ala-Arg-AMC substrate and r_s_ = 0.753, *p* < 0.001 for the Tos-Gly-Pro-Arg- AMC substrate, Figure 3H,I). No significant correlations were found for the other proteolytic activities (data not shown).

### 3.3. Fecal Trypsin-Like Activity Does Not Differ between Post-Colitis Rats and Controls

**Figure 4:**
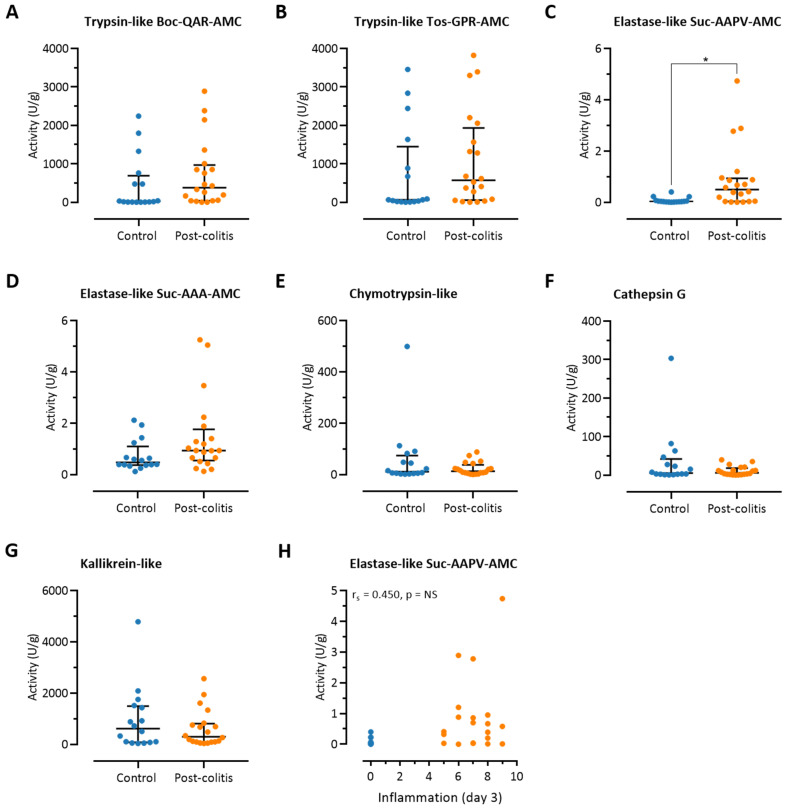
Proteolytic activities in fecal samples of post-colitis and control rats. (**A**) Trypsin-like activity assessed using Boc-Gln-Ala-Arg-AMC. (**B**) Trypsin-like activity assessed using Tos-Gly-Pro-Arg-AMC. (**C**) Elastase-like activity assessed using Suc-Ala-Ala-Pro-Val-AMC. (**D**) Elastase-like activity assessed using Suc-Ala-Ala-Ala-AMC. (**E**) Chymotrypsin-like activity. (**F**) Cathepsin G activity. (**G**) Kallikrein activity. Proteolytic activities were analyzed using Mann–Whitney U tests with Bonferroni correction for multiplicity; *n* = 16–20 per group. Data are presented including the median and IQR. (**H**) Correlation between the severity of inflammation in the acute phase of colitis (day 3) and elastase-like activity using Suc-Ala-Ala-Pro-Val-AMC. Correlations were assessed using Spearman’s rank order correlation coefficient, employing the Holm–Bonferroni method to correct for multiplicity. Note: * *p* < 0.05, significantly different from control. NS, non-significant.

Given that proteases in the lumen of the gut do not only originate from the colonic mucosa but can also be produced by the pancreas and the microbiome, fecal proteolytic activities were assessed. As opposed to the colonic samples, no differences were observed between post-colitis and control animals with regard to trypsin-like activity (Boc-Gln-Ala-Arg-AMC: 381.46 (50.63–891.71) U/g versus 24.37 (5.90–547.00) U/g, *p* = NS, Tos-Gly-Pro-Arg-AMC: 571.95 (72.36–1687.43) U/g versus 65.04 (19.85–1073.75) U/g, *p* = NS, *n* = 16–20 per group, pooling of different treatment groups due to pre-treatment collection of fecal pellets, Figure 4A,B). However, elastase-like activity measured with Suc-Ala-Ala-Pro-Val-AMC was significantly increased in fecal samples of post-colitis animals (0.49 (0.04–0.90) U/g versus 0.03 (0.02–0.05) U/g, *n* = 16–20 per group, *p* < 0.01, pooling of different treatment groups due to pre-treatment collection of pellets, Figure 4C). The host proteases with elastase-like activity [20,33] and the bacterial serine proteases EatA (*Escherichia coli*) [34] or subtilisin DY (*Bacillius licheniformis*) [35] could be responsible for the cleavage of this substate. Other specific serine protease activities in the fecal samples did not differ between post-colitis and control animals (Figure 4D–G). Elastase-like activity measured with Suc-Ala-Ala-Pro-Val-AMC correlated with the degree of inflammation at day 3, as measured by colonoscopy (r_s_ = 0.450, *p* = 0.006). However, after performing a Grubbs’s test to formally assess whether outliers were present, we concluded that the animal with the highest activity (4.74 U/g activity) could be considered an outlier. After removing this outlier, the correlation was no longer significant after correction for multiplicity with the Holmberg–Bonferroni procedure (r_s_ = 0.404, *p* = 0.016, *n* = 35, with a *p*-value considered to be statistically significant when *p* < 0.007, Figure 4H). For the other proteolytic activities, no significant correlations were found.

### 3.4. The Serine Protease Inhibitor UAMC-00050 Inhibits Trypsin-Like Enzymes In Vitro

**Figure 5:**
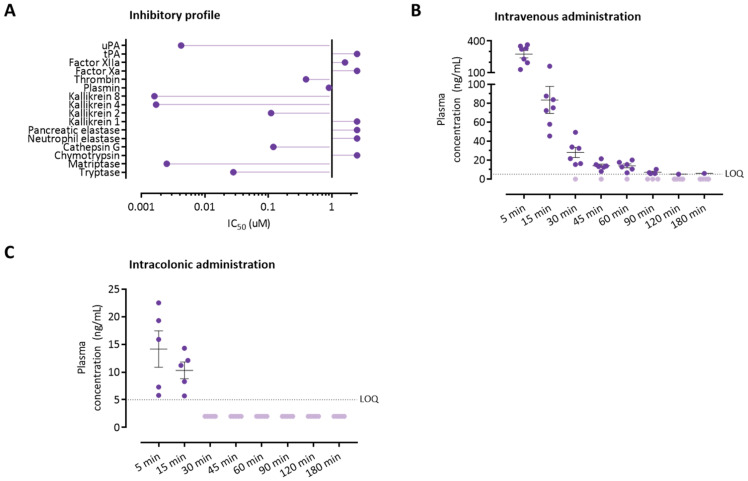
Pharmacokinetic properties of UAMC-00050. (**A**) Inhibitory profile of UAMC-00050 for a selection of proteases. IC_50_ > 2.5 µM, indicating non-efficient inhibition of the enzyme by UAMC-00050, are visualized as IC_50_ = 2.5 µM to allow for easier interpretation of the data. IC_50_ values were, in part, reproduced from Ceuleers and colleagues, with permission of the authors [16]. (**B**) Plasma concentrations after intravenous administration of UAMC-00050 (1 mg/kg), longitudinally collected, *n* = 7. Data are presented including means ± SEM. (**C**) Plasma concentrations after intracolonic administration of UAMC-00050 (5 mg/kg), longitudinally collected, *n* = 5. Data are presented including means ± SEM. LOQ, lower limit of quantification.

We previously described that UAMC-00050 efficiently inhibits trypsin-like enzymes such as uPA; plasmin; thrombin; matriptase; tryptase; cathepsin G; and kallikreins 2, 4, and 8. Furthermore, the inhibitor did not efficiently inhibit neutrophil elastase [16]. Here, the potency of UAMC-00050 to inhibit two other serine proteases, chymotrypsin and pancreatic elastase, with specificity was determined. IC50 values above 10 µM were obtained for both enzymes, suggesting no efficient inhibition and confirming the selectivity for trypsin-like proteases. The inhibitory profile of UAMC-00050 is summarized in Figure 5A.

### 3.5. Colonically Administered UAMC-00050 Is Not Detectable in Plasma after 30 Min

The pharmacokinetic properties of UAMC-00050 were determined after intravenous (1 mg/kg) or intracolonic (5 mg/kg) administration. After i.v. injection, UAMC-00050 followed a two-compartment model (Figure 5B, Table S4) with rapid distribution to the peripheral compartment (t_1/2,α_ = 5.8 min, *k_12_* = 0.028 min^−1^). UAMC-00050 redistributed from the peripheral to the central compartment at a slower rate (t_1/2,β_ = 66.5 min, *k*_21_ = 0.014 min^−1^).

Plasma concentrations of UAMC-00050 were low 5 and 15 min after intracolonic administration (14.1 ± 2.9 ng/mL and 10.3 ± 1.3 ng/mL, respectively, Figure 5C). UAMC-00050 was below the lower limit of quantification at later timepoints (30–180 min after administration). Five minutes after intracolonic administration, the plasma concentration of UAMC-00050 was on average only 5.4% of the plasma concentration after intravenous administration with a dose 5 times as low (5 mg/kg intracolonic versus 1 mg/kg intravenous), suggesting that UAMC-00050 stays in the lumen or colonic mucosa. No side-effects were observed within 180 min after the administration of the drug in rats.

### 3.6. Intracolonic Administration of UAMC-00050 Alters Trypsin-Like Activity in the Colon of Post-Colitis Animals

**Figure 6:**
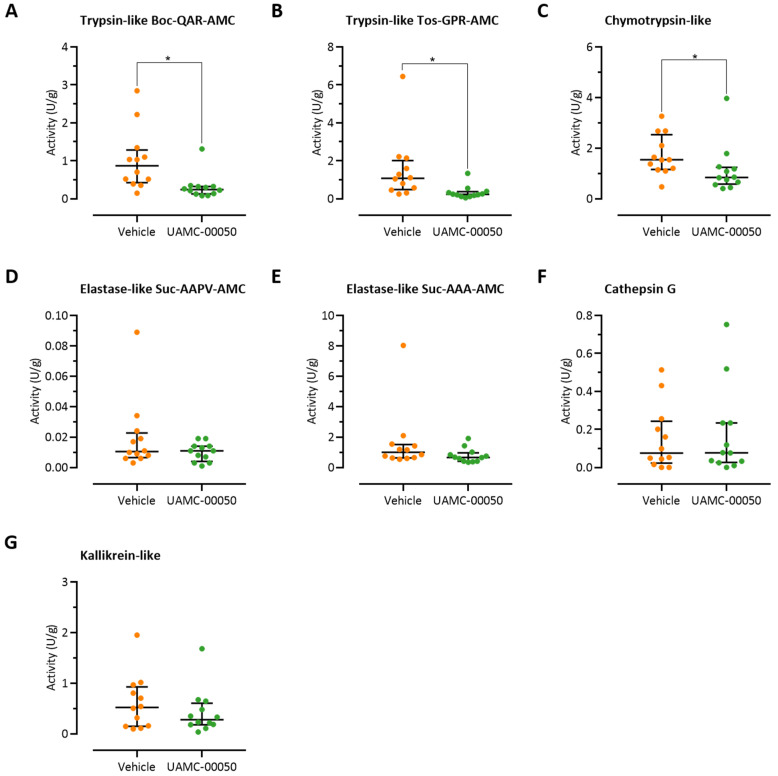
Proteolytic activities in colonic tissue of post-colitis rats treated with UAMC-00050 (5 mg/kg) or vehicle (5% DMSO). (**A**) Trypsin-like activity assessed using Boc-Gln-Ala-Arg-AMC. (**B**) Trypsin-like activity assessed using Tos-Gly-Pro-Arg-AMC. (**C**) Chymotrypsin-like activity. (**D**) Elastase-like activity assessed using Suc-Ala-Ala-Pro-Val-AMC. (**E**) Elastase-like activity assessed using Suc-Ala-Ala-Ala-AMC. (**F**) Cathepsin G activity. (**G**) Kallikrein activity. Proteolytic activities were analyzed using two-way ANOVA with ‘treatment’ and ‘group’ factors, followed by Mann–Whitney U tests with Bonferroni correction for multiplicity; *n* = 12 per group. Analysis was performed on the full dataset (involving the 4 combinations of control/post-colitis and vehicle/UAMC-00050), however visualization is split across Figure 2 and Figure 5, Figure S1 to allow for easier interpretation of the data. Data are presented including the median and IQR. Note: * *p* < 0.05; significantly different from post-colitis + vehicle.

Serine protease activity profiles were determined on colonic samples harvested 90 min after the intracolonic administration of UAMC-00050 (5 mg/kg). In post-colitis animals, a single administration of the serine protease inhibitor significantly decreased trypsin-like activity compared to vehicle-treated animals (Boc-Gln-Ala-Arg-AMC: 0.24 (0.87) U/g versus 0.87 (0.67) U/g, *p* < 0.01, Tos-Gly-Pro-Arg-AMC: 0.23 (0.18) U/g versus 1.08 (1.19) U/g, *p* < 0.01, *n* = 12 per group, Figure 6A,B). Moreover, treatment with UAMC-00050 resulted in lower chymotrypsin-like activity in the colon of post-colitis animals (0.84 (0.57) U/g versus 1.54 (1.06) U/g, *p* < 0.05, *n* = 12 per group, Figure 6C). No effect was seen for the other specific proteolytic activities (Figure 6D–G). UAMC-00050 did not alter proteolytic activities in colonic tissue of control animals (Figure S1).

### 3.7. Intracolonic Administration of UAMC-00050 Decreases Visceral Hypersensitivity in Post-Colitis Rats

**Figure 7:**
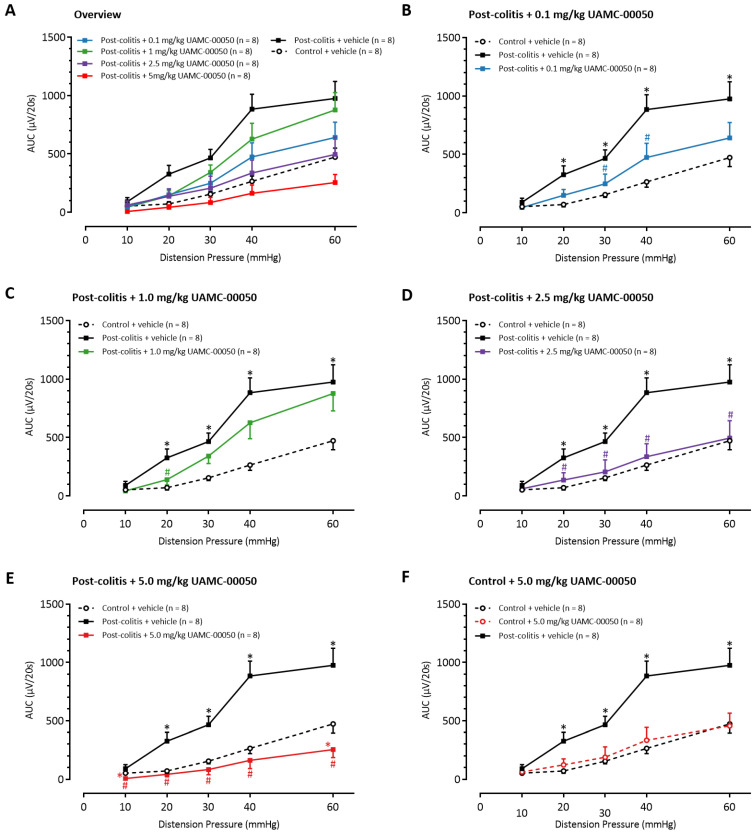
The effects of intracolonic administration of UAMC-00050 (0.1–1.0–2.5–5.0 mg/kg) and its vehicle (5% DMSO in H_2_O) on visceral sensitivity in post-colitis (filled squares) and control (open circles) rats. Statistical analysis was performed on the complete dataset (**A**), however separate graphs (**B**–**F**) were generated for each dose for the purpose of clarification. Data are presented as means ± SEM. Generalized estimating equations using an unstructured working correlation matrix, followed by a least significant difference (LSD) post hoc test; *n* = 8 per group. Note: * *p* < 0.05; significantly different from control + vehicle. Note: ^#^ *p* < 0.05; significantly different from post-colitis + vehicle.

Compared to control animals, vehicle-treated post-colitis rats showed significantly higher VMRs for distension pressures between 20 and 60 mmHg, indicating the presence of post-inflammatory visceral hypersensitivity (Figure 7A). A single intracolonic administration with UAMC-00050 in a low dose of 0.1 mg/kg attenuated visceral sensitivity at 30 and 40 mmHg (Figure 7B), whereas 1.0 mg/kg UAMC-00050 decreased visceral sensitivity at 20 mmHg (Figure 7C). No significant differences in the VMRs were detectable between the groups receiving 0.1 mg/kg or 1.0 mg/kg UAMC-00050. Administration of higher doses of UAMC-00050 resulted in further decreases of VMRs. Intracolonic treatment with 2.5 mg/kg UAMC-00050 completely restored sensitivity to normal values (Figure 7D). Remarkably, post-colitis rats treated with 5.0 mg/kg UAMC-00050 reached VMRs that were even lower than those in control rats (Figure 7E). In control rats however, no changes in visceral sensitivity were observed when the most effective dose of 5.0 mg/kg UAMC-00050 was administered in the colon (Figure 7F).

### 3.8. Intracolonic Administration of UAMC-00050 Increases Colonic Compliance

**Figure 8:**
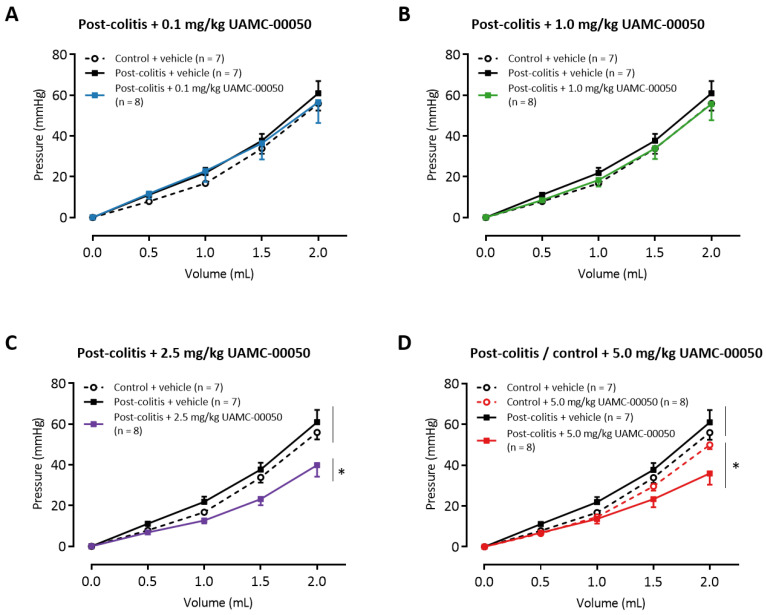
The effects of intracolonic administration of UAMC-00050 (0.1 (**A**)–1.0 (**B**)–2.5 (**C**)–5.0 (**D**) mg/kg) and its vehicle (5% DMSO in H_2_O) on colonic compliance in post-colitis (filled squares) and control (open circles) rats. Statistical analysis was performed on the complete dataset, however separate graphs were generated for each dose for the purpose of clarification. Data are presented as mean ± SEM. Generalized estimating equations using an unstructured working correlation matrix, followed by a least significant difference (LSD) post hoc test; *n* = 7–8 per group. Note: * *p* < 0.05; significantly different from vehicle treated animals.

Vehicle-treated post-colitis animals displayed a colonic compliance similar to control animals (Figure 8A). Intracolonic treatment of the animals with low doses of UAMC-00050 (0.1 mg/kg–1.0 mg/kg) did not alter colonic compliance (Figure 8A,B). Compared to vehicle-treated animals, a single treatment of animals with 2.5 mg/kg UAMC-00050 (Figure 8C) or 5.0 mg/kg UAMC-00050 (Figure 8D) resulted in lower intracolonic pressures when graded volumes of water were applied. Hence, colonic compliance is increased in these animals.

### 3.9. Intracolonic Administration of UAMC-00050 Increases MPO Activity in the Distal Colon Without Affecting Other Inflammatory Parameters

A single intracolonic administration of UAMC-00050 increased colonic MPO activity, both in control and post-colitis animals. There was no effect of the treatment with UAMC-00050 on other inflammatory parameters, i.e., colonoscopic inflammatory scores and macroscopic or microscopic evaluations of mucosal damage (Table 2).

## 4. Discussion

Visceral hypersensitivity is considered an important mechanism underlying abdominal pain in GI diseases such as IBD and IBS [36]. Serine proteases have been proposed as therapeutic targets for the treatment of visceral pain in IBS patients [1,15]. However, clinical studies with serine protease inhibitors are lacking due to concerns about systemic side effects, such as the risk of bleeding [1,37]. In our study, we demonstrated that a locally administered serine protease inhibitor improves dysregulated proteolytic activities in the colon of TNBS-induced post-inflammatory rats. Next, we showed that UAMC-00050 acts locally, being taken up in the bloodstream only minimally and for a limited amount of time after intracolonic administration, thus limiting the possibility of systemic side effects. Finally, we observed a normalization of post-inflammatory visceral hypersensitivity after local administration of UAMC-00050, supporting both the essential role of serine proteases in the pathophysiology of visceral pain and the potential of locally administered serine protease inhibitors as clinically relevant compounds for the treatment of IBS patients with abdominal pain.

Early studies in IBS suggested an increased fecal proteolytic activity compared to healthy volunteers, with a potential role for serine proteases in IBS-D [5,9,10] and cysteine proteases in IBS-C [38]. Which specific proteases form the basis of the increased proteolytic activity remained elusive due to the lack of well-characterized activity-based probes or fluorogenic substrates for detecting the activity of the serine proteases and not just their expression [39]. In this study, we used selective substrates to further unravel the activity profile of the serine proteases involved. Whereas alterations in the fecal proteolytic profile were not detectable in post-colitis rats in our previous study [16], the larger sample size of the current study allowed us to observe a significant increase in elastase-like activity with Suc-AAPV-AMC in the fecal samples of post-colitis rats but not in colonic tissue, whereas trypsin-like activities were increased in colonic samples but not in fecal pellets.

Trypsin has been proposed as a therapeutic target for functional GI disorders such as IBS [1], however to our knowledge no specific trypsin inhibitors have been developed. Increased expression of trypsin [8,40] and a greater colonic trypsin-like activity [12,41] has been found in multiple cohorts of IBS patients, comprising patients of several IBS subtypes. In agreement with findings of our previous study [16], the trypsin-like activity was increased in the post-inflammatory rats compared to controls, which was demonstrated using two different substrates for trypsin-like enzymes. Moreover, we show for the first time that the serine protease inhibitor UAMC-00050 inhibits colonic trypsin-like activity after intracolonic administration. Intriguingly, trypsin-like activity in the post-inflammatory state correlates with the initial degree of inflammation during acute colitis. Hence, we hypothesize that the initial degree of inflammation results in an alteration of the proteolytic profile that persists in the post-inflammatory state. Although UAMC-00050 does not inhibit chymotrypsin-like activity in vitro, we noted a decrease of chymotrypsin-like activity after the administration of UAMC-00050 in vivo. Chymotrypsin is secreted from the pancreas as a zymogen and is activated by trypsin in the digestive tract [42]. Potentially, the decreased trypsin-like activity in animals treated with UAMC-00050 resulted in less activation of chymotrypsin. Additionally, UAMC-00050 did not decrease trypsin-like activity in control rats. We hypothesize that only a subset of trypsin-like enzymes is increased in the post-colitis animals, and that the inhibitory effect of UAMC-00050 is limited to this specific set of proteases. However, the individual trypsin-like enzymes involved remain to be identified.

Elastase-like activity was also found to be increased in post-colitis animals, albeit only in the fecal samples. This increase can be due to an increased activity of the host’s protease [20,33] or bacterial enzymes from *E. coli* [34] or *B. licheniformis* [35]. Neutrophil elastase is passively released or actively secreted by neutrophils, and might be an important regulatory enzyme linked with inflammatory conditions such as inflammatory bowel disease [43,44,45]. Its increased luminal or fecal but not mucosal activity might indicate that micro-inflammation is still present in the post-colitis rats, although other inflammatory parameters (colonoscopy, histological examination, and MPO activity) were normalized on the day of VMR experiments. Increased elastase activity in fecal samples was previously shown to differentiate mild from severe DSS-induced murine colitis, as well as patients with active ulcerative colitis from patients in remission [46]. Hence, it might be used as a marker of microscopic residual inflammation. Edogawa and colleagues [13] recently characterized proteolytic activities in fecal supernatants of IBS patients and identified greater activity of elastase in a subpopulation of patients with increased proteolytic activity compared to healthy controls, which is in line with our findings. However, we did not observe the increased trypsin-like and chymotrypsin-like activities in the fecal samples that were observed in their study population.

Being one of the main contributors to luminal proteolytic activity, the pancreas has been a recent focus of investigations in the IBS field [10,11,13]. In a pivotal study, Tooth and colleagues pointed out that the presence of increased protease activity in the gut lumen of IBS patients is most likely due to pancreatic enzymes [11], although they only detected alterations in the activity of the starch-hydrolyzing amylase in a subpopulation of patients, with no differences in fecal elastase activity. Although the pancreas was not our main focus of our study, we could not detect differences in trypsin-like and elastase-like activity, as measured with Suc-Ala-Ala-Ala-AMC, between post-inflammatory and control animals in fecal samples.

Characterization of proteolytic profiles in clinical studies supports the existence of increased proteolytic activity in only a subpopulation of IBS patients [11,13]. Moreover, greater proteolytic activity might be due to the activity of distinct proteases or even protease classes (e.g., serine proteases and cysteine proteases), as shown by our results and the existing literature [5,13,38]. We already emphasized the different sources of these increased proteolytic activities, including the gut epithelium, the pancreas, and the microbiome. The differences in proteolytic activities might, thus, be driven by the microbiome or host–microbiome interactions. Indeed, a decreased microbial diversity and changes in the composition of the fecal microbiome, have been described in patients with greater proteolytic activity in the intestinal lumen [13]. However, further research is necessary to identify which microbial species associate with increased proteolytic activities and which host-related factors are involved in its regulation. Furthermore, the development of clinical assays to select patients who might be responsive to treatment with specific protease-modulating therapeutics is of great importance. Ideally, these assays should focus on detecting functional proteolytic activity profiles instead of on the expression of proteases [23].

Although no clinical data are currently available, several experimental studies point towards the antinociceptive effects of serine protease inhibitors in an acute restraint stress-induced rat model for IBS [47] and in murine models for visceral hypersensitivity using biopsy [8] or a fecal [48] supernatant of IBS patients. We previously demonstrated antinociceptive effects of UAMC-00050 in TNBS-induced post-inflammatory rats after intraperitoneal administration [16]. In this study, we confirmed that these effects were equally present after intracolonic administration, although a higher dose was required to obtain complete reversal of visceral hypersensitivity (2.5 mg/kg intracolonic versus 1.0 mg/kg intraperitoneally). Several reasons might explain this difference. First, the necessity of a higher dose could be due to a lower bioavailability of the compound at the level of the colonic mucosa after intracolonic administration. However, preliminary experiments with similar volumes of a solution containing the Evans Blue dye showed an even staining of the dye in the distal colon of the rat and no expulsion of the enema. Second, pharmacokinetic experiments showed a limited uptake of UAMC-00050 in the serum after intracolonic administration, indicating poor transport across the colorectal wall, and therefore suggesting that UAMC-00050 acts locally. However, we cannot exclude that UAMC-00050 is metabolized in the colon, and therefore is not detected in the serum. The detailed mechanism of action of UAMC-00050 remains to be elucidated, and so far we cannot completely exclude that UAMC-00050 has systemic effects.

Local administration of UAMC-00050 revealed some surprising effects on colonic tissue. First, we observed an increase in colonic compliance when a high dose of UAMC-00050 (2.5–5 mg/kg) was administered. Colonic compliance contributes to the sensation of gas and pain in response to colorectal distensions [49], and therefore might have altered the response of our rats to colorectal distensions. Interestingly, clinical studies have shown that increased compliance is associated with increased sensation in healthy volunteers [49,50]. However, whether this relationship extends to non-physiological conditions such as IBD [51] or IBS [52,53] still has to be studied in more detail. The mechanism underlying the observed alterations in colonic compliance requires further investigation. Among the possibilities are alterations in the connective tissue matrix or smooth muscle tone [54]. Nonetheless, the significant effect of a low dose of UAMC-00050 (0.1–1 mg/kg) on visceral pain without an accompanying effect on the viscoelastic properties of the colon suggests a direct effect of the compound on pain signaling pathways, which is in line with our previous findings [16].

Besides the effects of UAMC-00050 on colonic compliance, a small but significant increase in MPO activity was seen in colonic tissue samples of both post-colitis and control rats. MPO activity is a measure of myeloid cell infiltration [31]. Interestingly, UAMC-00050 showed anti-inflammatory effects in a murine CD4^+^CD25^−^CD62L^+^ T-cell transfer model for chronic colitis [55] and a rat model of dry eye syndrome [27]. Moreover, no effects on inflammatory parameters were seen after intraperitoneal treatment with the same compound in the post-inflammatory model [16]. The explanation for the increased MPO activity after local administration of UAMC-00050 is not clear at this moment; it might be due to the disturbance of the protease–anti-protease balance interfering with inflammatory or anti-inflammatory mediators or due to its interaction with other luminal components (e.g., the microbiome or its metabolites).

Taken together, our results indicate that the local administration of a serine protease inhibitor can modulate proteolytic activities in vivo and reverse visceral hypersensitivity while acting locally. Hence, this study paves the way towards a locally acting therapeutic approach for serine protease inhibitors in gastrointestinal disorders such as IBS.

## Figures and Tables

**Table 1:** Optimized compound-specific mass spectrometric parameters for pharmacokinetic experiments.

Compound	Retention Time (min)	Precursor Ion (*m*/*z*)	Product Ion (*m*/*z*)	Fragmentor Voltage (V)	Collision Energy (eV)
UAMC-00050	6.3	659.2	**91.2**	185	60
			267.3	185	40
			250.2	185	35
Nordazepam-D_5_	7.0	276.0	**140.0**	100	35

The quantifier transition is indicated in bold.

**Table 2:** Inflammatory parameters in the colon of post-colitis and control rats.

Group	Drug	N	Day 3	Day of VMR			
			Colonoscopy	Colonoscopy	Macroscopy	Microscopy	MPO Activity
Control	Vehicle	8	0.0 ± 0.0	0.0 ± 0.0	0.0 ± 0.0	0.5 ± 0.3	0.3 ± 0.1
	5.0 mg/kg	8	0.0 ± 0.0	0.0 ± 0.0	0.0 ± 0.0	0.8 ± 0.4	0.6 ± 0.2 ^#^
Post-colitis	Vehicle	8	6.3 ± 0.6 *	0.0 ± 00	0.0 ± 0.0	0.6 ± 0.4	0.3 ± 0.2
	0.1 mg/kg	8	7.5 ± 0.5 *	0.0 ± 0.0	0.0 ± 0.0	0.4 ± 0.4	2.0 ± 0.7 ^#^
	1.0 mg/kg	8	7.4 ± 0.6 *	0.0 ± 0.0	0.0 ± 0.0	0.8 ± 0.4	2.0 ± 0.5 ^#^
	2.5 mg/kg	8	7.1 ± 0.5 *	0.0 ± 0.0	0.0 ± 0.0	0.5 ± 0.4	2.1 ± 0.5 ^#^
	5.0 mg/kg	8	6.9 ± 0.5 *	0.0 ± 0.0	0.0 ± 0.0	0.6 ± 0.4	1.7 ± 0.5 ^#^

Data are presented as means ± SEM. Two-Way ANOVA followed by Student–Newman–Keuls post hoc test; *n* = 8 per group. No significant interaction between the ‘group’ and ‘treatment’ factors. Note: * *p* < 0.05; significant effect of the ‘group’ factor. Note: ^#^ *p* < 0.05; significant effect of the ‘treatment’ factor. MPO, myeloperoxidase; N, number; VMR, visceromotor response.

## Data Availability

The data presented in this study are available on request from the corresponding author.

## Supplementary Materials

The following are available online at https://www.mdpi.com/article/10.3390/pharmaceutics13060811/s1: Figure S1: Proteolytic activities in colonic tissue of control rats treated with UAMC-00050 (5 mg/kg) or vehicle (5% DMSO). Table S1: Scoring system for inflammation—colonoscopy. Table S2: Scoring system for inflammation—macroscopy. Table S3: Scoring system for inflammation—microscopy. Table S4. Pharmacokinetic parameters.
