# Supplementary material for: Local Colonic Administration of a Serine Protease Inhibitor Improves Post-Inflammatory Visceral Hypersensitivity in Rats
Source: Pharmaceutics. 2021 May 29;13(6):811. doi: 10.3390/pharmaceutics13060811 (PMC8229129; doi:10.3390/pharmaceutics13060811)
Supplement: Supplementary file 1 [file pharmaceutics-13-00811-s001.zip › pharmaceutics-1175106-supplementary.pdf]

pharmaceutics

MDPI

# Supplementary Materials: Local Colonic Administration of a Serine Protease Inhibitor Improves Post-Inflammatory Visceral Hypersensitivity in Rats

**Nikita Hanning, Michelle De bruyn, Hannah Ceuleers, Tim Boogaerts, Maya Berg, Annemieke Smet, Heiko U. De Schepper, Jurgen Joossens, Alexander L. N. van Nuijs, Joris G. De Man, Koen Augustyns, Ingrid De Meester and Benedicte Y. De Winter**

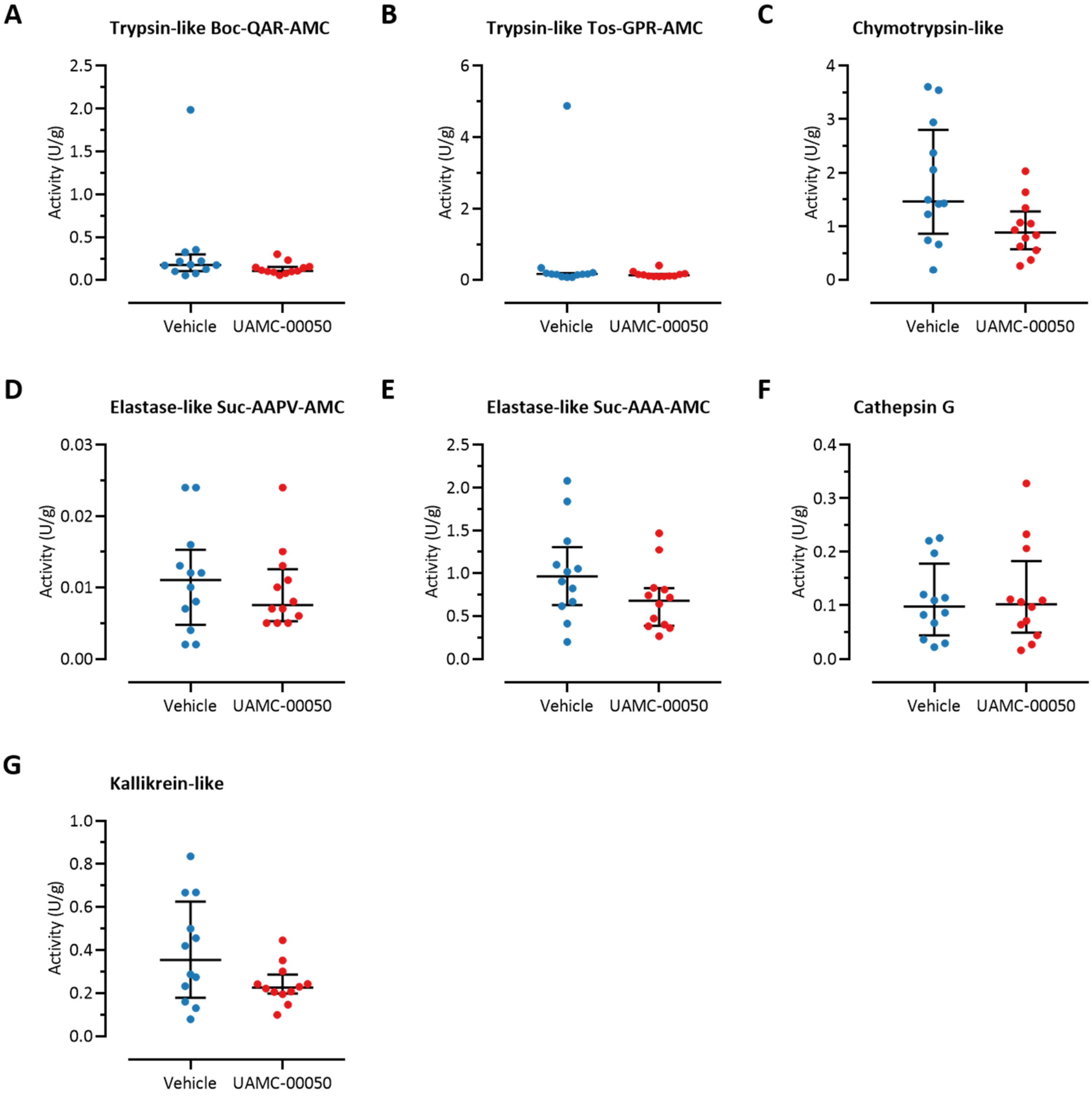


**Figure S1.** Proteolytic activities in colonic tissue of control rats treated with UAMC-00050 (5 mg/kg) or vehicle (5% DMSO). (**a**) Trypsin-like activity using Boc-Gln-Ala-Arg-AMC. (**b**) Trypsin-like activity using Tos-Gly-Pro-Arg-AMC. (**c**) Chymotrypsin-like activity. (**d**) Elastase-like activity using Suc-Ala-Ala-Pro-Val-AMC. (**e**) Elastase-like activity using Suc-Ala-Ala-Ala-AMC. (**f**) Cathepsin G activity. (**g**) Kallikrein activity. Proteolytic activities were analysed using two-way ANOVA with factors 'treatment' and 'group', followed by Mann-Whitney U tests with Bonferroni correction for multiplicity; n = 12 per group. Analysis was performed on the full dataset (involving the 4 combinations of control/post-colitis and vehicle/UAMC-00050), but visualization is split across Figure 2, Figure 5 and Supplementary Figure 1 to allow for easier interpretation of the data. Data are presented including median and IQR.

**Table S1.** Scoring system for inflammation – colonoscopy.

| Item | Aspect | Score |
|---|---|---|
| Ulceration | Normal mucosa | 0 |
| | Hyperemia | 1 |
| | Ulceration(s) occupying < 25% of the lumen circumference | 2 |
| | Ulceration(s) occupying 25 – 50% of the lumen circumference | 3 |
| | Ulceration(s) occupying > 50% but not the full circumference | 4 |
| | Ulceration(s) occupying the full circumference | 5 |
| | Ulceration(s) occupying the full circumference with longitudinal extension | 6 |
| Stenosis | Absent | 0 |
| | Present | 1 |
| Edema | Absent | 0 |
| | Present | 1 |
| Bleeding | Absent | 0 |
| | Present | |
| Extent of damage | Range 0 – 10 cm | 0 – 10 |
| **Total damage score** | | **0 – 19** |

**Table S2.** Scoring system for inflammation – macroscopy.

| Macroscopic Finding | Score |
|---|---|
| Normal mucosa | 0 |
| Localized hyperemia | 1 |
| Ulceration(s) without hyperemia or bowel wall thickening | 2 |
| Ulceration(s) with hyperemia or bowel wall thickening at 1 site | 3 |
| Ulceration(s) with hyperemia or bowel wall thickening at 2 or more sites | 4 |
| Major sites of damage extending up to 1 cm along the length of the colon | 5 |
| Major sites of damage extending up > 1 cm along the length of the colon | 6 – 10 |
| **Total damage score** | **0 – 10** |

**Table S3.** Scoring system for inflammation – microscopy.

| Histopathological finding | Normal | Minimal | Mild | Maximal |
|---|---|---|---|---|
| Inflammatory infiltrate | 0 | 1 | 2 | 3 |
| Number of infiltrated layers | 0 | 1 | 2 | 3 |
| Mucosal tissue damage | 0 | 1 | 2 | 3 |
| Mucosal edema | absent = 0; present = 1 | | | |
| **Total damage score** | **0 – 10** | | | |

**Table S4.** Pharmacokinetic parameters.

| Parameter | Unit | Value |
|---|---|---|
| A | ng/mL | 500.7 |
| Alpha | 1/min | 0.119 |
| B | ng/mL | 17.9 |
| Beta | 1/min | 0.010 |
| k10 | 1/min | 0.087 |
| k12 | 1/min | 0.028 |
| k21 | 1/min | 0.014 |
| t1/2Alpha | min | 5.800 |
| t1/2Beta | min | 66.5 |
| C0 | ng/ml | 518.7 |
| V | (mg)/(ng/mL) | 0.006 |
| CL | (mg)/(ng/mL)/min | 0.000507175 |
| V2 | (mg)/(ng/mL) | 0.011 |

| | | |
|---|---|---|
| CL2 | (mg)/(ng/mL)/min | 0.000162136 |
| AUC 0-t | ng/mL*min | 5422 |
| AUC 0-inf | ng/mL*min | 5915 |
| AUMC | ng/mL*min$^2$ | 200,489 |
| MRT | min | 33.9 |
| Vss | mg/(ng/mL) | 0.017 |
